# The Adverse Effect of Weight Stigma on the Well-Being of Medical Students with Overweight or Obesity: Findings from a National Survey

**DOI:** 10.1007/s11606-015-3266-x

**Published:** 2015-07-15

**Authors:** Sean M. Phelan, Diana J. Burgess, Rebecca Puhl, Liselotte N. Dyrbye, John F. Dovidio, Mark Yeazel, Jennifer L. Ridgeway, David Nelson, Sylvia Perry, Julia M. Przedworski, Sara E. Burke, Rachel R. Hardeman, Michelle van Ryn

**Affiliations:** 1 College of Medicine, Mayo Clinic, 200 First Street SW, Rochester, MN 55905 USA; 2 Center for Chronic Disease Outcomes Research, Minneapolis VAMC, Department of Medicine, University of Minnesota, Minneapolis, MN USA; 3 Rudd Center for Food Policy & Obesity, University of Connecticut, Hartford, CT USA; 4 Department of Psychology, Yale University, New Haven, CT USA; 5 Department of Family Medicine, University of Minnesota, Minneapolis, MN USA; 6 Mayo Clinic Division of Health Care Policy and Research, Rochester, MN USA; 7 Department of Psychological Science, University of Vermont, Burlington, VT USA; 8 Division of Health Policy and Management, University of Minnesota, Minneapolis, MN USA; 9 Department of Psychology, Yale University, New Haven, CT USA; 10 Mayo School of Medicine & Division of Health Care Policy and Research, Mayo Clinic, Rochester, MN USA

**Keywords:** Medical students, Stigmatization, Psychological stress, Obesity, Body weight

## Abstract

**BACKGROUND:**

The stigma of obesity is a common and overt social bias. Negative attitudes and derogatory humor about overweight/obese individuals are commonplace among health care providers and medical students. As such, medical school may be particularly threatening for students who are overweight or obese.

**OBJECTIVE:**

The purpose of our study was to assess the frequency that obese/overweight students report being stigmatized, the degree to which stigma is internalized, and the impact of these factors on their well-being.

**DESIGN:**

We performed cross-sectional analysis of data from the Medical Student Cognitive Habits and Growth Evaluation Study (CHANGES) survey.

**PARTICIPANTS:**

A total of 4,687 first-year medical students (1,146 overweight/obese) from a stratified random sample of 49 medical schools participated in the study.

**MAIN MEASURES:**

Implicit and explicit self-stigma were measured with the Implicit Association Test and Anti-Fat Attitudes Questionnaire. Overall health, anxiety, depression, fatigue, self-esteem, sense of mastery, social support, loneliness, and use of alcohol/drugs to cope with stress were measured using previously validated scales.

**KEY RESULTS:**

Among obese and overweight students, perceived stigma was associated with each measured component of well-being, including anxiety (beta coefficient [b] = 0.18; standard error [SE] = 0.03; *p* < 0.001) and depression (b = 0.20; SE = 0.03; *p* < 0.001). Among the subscales of the explicit self-stigma measure, dislike of obese people was associated with several factors, including depression (b = 0.07; SE = 0.01; *p* < 0.001), a lower sense of mastery (b = −0.10; SE = 0.02; *p* < 0.001), and greater likelihood of using drugs or alcohol to cope with stress (b = 0.05; SE = 0.01; *p* < 0.001). Fear of becoming fat was associated with each measured component of well-being, including lower body esteem (b = −0.25; SE = 0.01; *p* < 0.001) and less social support (b = −0.06; SE = 0.01; *p* < 0.001). Implicit self-stigma was not consistently associated with well-being factors. Compared to normal-weight/underweight peers, overweight/obese medical students had worse overall health (b = −0.33; SE = 0.03; *p* < 0.001) and body esteem (b = −0.70; SE = 0.02; *p* < 0.001), and overweight/obese female students reported less social support (b = −0.12; SE = 0.03; *p* < 0.001) and more loneliness (b = 0.22; SE = 0.04; *p* < 0.001).

**CONCLUSIONS:**

Perceived and internalized weight stigma may contribute to worse well-being among overweight/obese medical students.

To succeed academically and professionally, medical students must withstand the stress of medical school, including learning new and complex material, meeting faculty expectations, interacting with patients, making new friends and colleagues, and assimilating the culture of medicine.^1^–^3^ Ability to cope with stress is important to health and professional development, as medical student stress is linked to burnout, substance use, mental health problems, suicidal thoughts, and poor academic performance.^1^,^4^–^6^ Stress also disproportionately affects female medical students, who may then be more vulnerable to these outcomes.^5^,^7^–^9^

Self-esteem, physical and emotional health, fatigue, sense of mastery, and social support all affect vulnerability to stress.^10^,^11^ Members of stigmatized groups, including overweight/obese individuals, may face additional stress.^12^–^17^ Experiences of weight-related stigma can have negative effects on self-esteem, health, and well-being.^12^–^14^,^17^–^23^ Overweight/obese individuals may also be self-stigmatized, i.e., exhibit negative, self-deprecating attitudes about themselves, which may worsen their overall well-being.^24^–^26^

These additional stressors may challenge students’ ability to cope in the competitive medical school environment. Although little is known about the experience of these medical students, several studies have documented strong anti-fat attitudes among health care providers and trainees,^27^–^31^ and suggest that overweight/obese individuals are a common target of derogatory humor among medical students.^32^,^33^

The present study aimed to assess whether stigma or self-stigma is associated with factors that affect vulnerability to stress among overweight and obese medical students. We hypothesized that 1) these medical students, and female students in particular, have worse self-reported outcomes than normal-weight/underweight medical students on factors affecting vulnerability to stress; and that 2) among overweight/obese students, experiencing more stigma/self-stigma is associated with worse outcomes.

## METHODS

### Sample

This study used data from the Medical Student Cognitive Habits and Growth Evaluation Study (CHANGES), a study of first-year students at Liaison Committee on Medical Education (LCME)-accredited U.S. medical schools that was conducted in the fall of 2010. Our research study was approved by the institutional review board of the Mayo Clinic. We randomly selected 50 medical schools from the strata of public and private schools in six regions of the country, using sample-proportional-to-strata-size methodology. One sampled school with highly unique characteristics (military school) was excluded, leaving a sample of 49 schools. From these schools, we invited 5,823 first-year students (68 % of all first-year medical students attending sampled schools) to participate in a Web-based survey. We used a three-part ascertainment strategy (see Phelan et al.^27^). First, the AAMC inserted a question into their voluntary Matriculating Student Questionnaire, sent to all incoming students, asking respondents to provide an email address in order to learn more about our study. Second, we purchased a list of first-year students from an AMA-licensed vendor. Third, we asked survey respondents to send study information to their classmates. The sample (*n* = 4,732) comprised 81 % of those who were sent an invitation and 55 % of the entire pool of first-year medical students at study schools. Of the 4,732 students, 34 were excluded due to incomplete questionnaires or evidence of systematic response bias. Of the remaining 4,698 students, 4,687 had non-missing height and weight information.

### Data Collection and Integrity

Data were collected online between October and December 2010. All students completed the survey during their first semester of medical school. Students were sent an email with a link to an online questionnaire. If participants chose not to answer a question, they were required to click on a button to indicate their desire to skip the question. This protected the participant’s right to skip questions, while eliminating time-saving incentives for doing so. Students were randomly assigned to complete either the Weight Implicit Association Test (IAT), a measure of implicit bias, (*n* = 2,370), or an IAT measuring bias against a different stigmatized group. Participants received a $50.00 cash incentive.

## MEASURES

We used previously validated scales with satisfactory psychometric properties. Unless otherwise noted, scale variables are a mean of indicator items identified by principal component analysis. Scale variables were created if the respondent completed at least 60 % of items making up a scale. Except where noted below, items were measured on a 5-point scale. To limit respondent burden, subsets of items were selected from longer scales to represent specified subscales. Height and weight were self-reported and body mass index (BMI) was calculated and categorized as overweight/obese (BMI = 25 or higher) or normal/underweight (BMI = 24.9 or lower).

### Stress Vulnerability Factors

Self-esteem. Self-esteem was measured using items selected from all three subscales of the 20-item State Self-Esteem Scale: 1) *Confidence in Cognitive Abilities* (Cronbach’s alpha (α) = 0.83), consisting of four items, including “I feel as smart as others;” 2) *Body Esteem* (α = 0.80), consisting of three items, including “I am pleased with my appearance right now;” and 3) *Importance of Impression on Others* (α = 0.79), consisting of four items, including “I am worried about looking foolish.”^34^

Physical Health, Fatigue, and Emotional Health. Self-assessed physical health was measured using a single item from the Medical Outcomes Study Short Form Health Survey,^35^ “How is your overall health right now?” Responses ranged from “excellent” to “poor.” Anxiety (α = 0.92), depression (α = 0.94), and fatigue (α = 0.88) were measured using the Patient-Reported Outcomes Measurement Information System (PROMIS) short forms.^36^–^38^

Mastery. An individual’s sense of power and control over his/her life was measured using the seven-point Pearlin Mastery Scale (α = 0.82).^39^ Items included “I have little control over the things that happen to me.”

Social Support. We included measures of both social support and social isolation. Social support was measured using the 19-item Medical Outcomes Study Social Support Survey (α = 0.96).^40^ Items included “How often do you have someone to confide in or talk to about yourself.” Loneliness was measured using the three-item Brief UCLA Social Loneliness Scale (α = 0.84), including “How often do you feel left out?”^41^

Substance Use for Coping. The use of drugs or alcohol to cope with stress was measured as a single item from the Brief COPE measure.^42^ Participants indicated on a four-point scale how often they used drugs or alcohol to cope with stress.

### Stigma

Self-stigma. Items selected from Crandall’s Anti-Fat Attitudes Questionnaire were used to assess explicit self-stigma among overweight/obese students.^43^ This scale has three subscales: *1*) *Dislike* (α = 0.86), three items, e.g., “I really don’t like fat people much"; *2*) *Willpower* (α = 0.74), two items, e.g., “Fat people tend to be fat pretty much through their own fault”; and *3*) *Fear of Fat* (α = 0.79), two items, e.g., “I feel disgusted with myself when I gain weight.” All items were measured on a seven-point scale, from “strongly disagree” to “strongly agree.”

The Fat-Thin IAT was used to measure automatic self-stigma among overweight/obese students. This measure compares the length of time that respondents use to categorize images of fat and thin people with positive and negative words. The IAT has been extensively validated and shown to predict discriminatory behavior independently of explicit attitudes.^44^,^45^ IAT D (difference) scores (range: −2 to 2) were categorized according to commonly used cut points for slight (0.15–0.35), moderate (0.36–0.60), and strong (> 0.60) attitudes.

Perceived Stigma. Experiences of stigma were assessed using the nine-item Everyday Discrimination Scale (α = 0.87), which measures the frequency of perceived poor treatment.^46^ Examples of items include “In your day-to-day life, how often are you treated with less respect than other people?” Items in the six-point response scale range from “once a week or more” to “never.”

### Covariates

Race, gender, age, and relationship status were measured using standard questionnaire items. Race was categorized into students who self-identified as non-Hispanic white compared to all other race/ethnicity groups. Highest educational attainment of either parent (any graduate degree, college degree, or less than a college degree) was used as a marker for socioeconomic status. Social desirability bias was measured using seven items from the Marlowe-Crowne Social Desirability Index.^47^

### Analysis

All analyses were weighted to account for stratified sampling of schools. Associations between overweight/obese BMI and stress vulnerability factors were assessed in multivariate linear regression models, adjusted for gender, age, non-white race, relationship status, parents’ highest education, and social desirability. Students who were overweight or obese were categorized together in order to identify the largest group of students who were at risk and who might be targeted for supportive interventions. We then added interactions between BMI and gender to each model to test whether associations between BMI and stress vulnerability factors varied by gender. For each model where the interaction term was significant (*p* < 0.10), we evaluated the associations between BMI and stress vulnerability factors separately for men and women.

We calculated the adjusted mean perceived stigma and IAT scores for overweight/obese students, accounting for all covariates, and measured endorsement of items indicating explicit self-stigma.

We used multivariate linear regression models to assess the associations between self-stigma and each stress vulnerability factor among students who were overweight/obese. The traditional significance level of *p* < 0.05 was used for all analyses. Although with large samples, statistically significant effects at *p* < 0.05 may reflect relatively small effect sizes, small but systematic effects for the variables we studied can have a meaningful impact on mental health.

## Key Results

We invited 5,823 students (68 % of all first-year students attending sampled schools) to participate in the Web-based survey. Respondents (*n* = 4,732) comprised 81 % of those who were sent an invitation and 55 % of the entire pool of first-year students. The sample had gender and race distribution similar to the overall first-year student population among the study schools. Demographic characteristics of the sample are presented in Table 1. Three-fourths of the study sample had an underweight/normal-weight BMI (76 %, *n* = 3,541), and one-fourth had a BMI in the overweight/obese range (24 %, *n* = 1,146).

**Table 1:** Sample Characteristics for 4,687 First-Year Medical Students Participating in Medical Student CHANGES, a 2010 Web-Based Study of Students at 49 LCME-Accredited U.S. Medical Schools

	Sample % (n)
BMI category	
Underweight (BMI < 18.5)	3.5 % (163)
Normal-weight (BMI: 18.5 to 24.9)	72.1 % (3,378)
Overweight (BMI: 25.0 to 29.9)	19.7 % (922)
Obese (BMI ≥ 30)	4.8 % (224)
Age	23.9 (2.6)
Female	50.0 % (2,344)
Race/ethnicity	
Non-white	36.7 % (1,689)
Non-Hispanic white	63.3 % (2,913)
Parents’ highest education	
Graduate degree	62.8 % (2,938)
College degree	21.8 % (1,023)
No college degree	15.4 % (721)
Relationship status	
Married, engaged, or cohabiting	16.9 % (791)
Not in a relationship or in a relationship but not cohabiting	83.1 % (3,896)

### Well-being/Stress Vulnerability Factors

Compared to underweight/normal-weight students, overweight/obese students reported significantly worse overall health, body esteem, and loneliness, and were more likely to report using alcohol or drugs to cope with stress (Table 2).

**Table 2:** Descriptive Statistics and Associations Between Obese/Overweight BMI and Stress Vulnerability Factors in the Medical Student CHANGES Study, a 2010 Web-Based Survey Study of 4,687 First-Year Medical Students from 49 LCME-Accredited U.S. Medical Schools

Measure (scale range)	Mean^*^ obese/overweight (BMI ≥ 25) *n* = 1,146	Mean^*^ normal/underweight (BMI < 25) *n* = 3,541	Beta coefficient (standard error)^a^	p value	p value for BMI category*gender interaction
Overall health (1–5)	3.49	3.82	−0.33 (0.03)	<0.001^†^	0.05
Anxiety symptoms (1–5)	2.61	2.59	0.03 (0.03)	0.46	0.68
Depression symptoms (1–5)	1.80	1.83	−0.03 (0.03)	0.24	0.17
Fatigue symptoms (1–5)	2.88	2.85	0.04 (0.03)	0.23	0.49
State self-esteem: confidence in cognitive abilities subscale (1–5)	3.54	3.55	−0.01 (0.02)	0.45	0.31
State self-esteem: body esteem subscale (1–5)	2.83	3.52	−0.70 (0.02)	<0.001	<0.001
State self-esteem: impression on others subscale (1–5)	3.24	3.26	−0.03 (0.03)	0.35	0.97
Mastery (1–7)	5.68	5.64	0.04 (0.03)	0.09	0.08
Social Support (1–5)	4.08	4.11	−0.03 (0.03)	0.23	0.002
Loneliness (1–5)	2.40	2.35	0.05 (0.02)	0.03	<0.001
Use of alcohol/drugs to cope (1–4)	1.52	1.45	0.07 (0.02)	0.007	0.32

* Means and parameter estimates adjusted for age, gender, relationship status, non-white race, parents’ highest education, and social desirability

^†^ *P* values from Wald F tests

Higher scale scores represent more of the concept being measured

They did not report greater anxiety symptoms or depressive symptoms, and did not have lower overall trait self-esteem or sense of mastery.

In assessing the interaction between BMI category and gender, we found a significant difference in body esteem, social support, and loneliness, and borderline significance in overall health and sense of mastery. Women who were overweight/obese had significantly worse overall health, body esteem, and social support and had higher mean loneliness scores than their normal-weight/underweight counterparts. Among male students, similar findings emerged for overall health and body esteem in the overweight/obese versus normal/underweight group. Male students who were overweight/obese had a greater sense of mastery than normal/underweight men (Table 3).

**Table 3:** Associations Between Obese/Overweight BMI and Stress Vulnerability or Resiliency Factors by Gender in the Medical Student CHANGES, a 2010 Web-Based Survey Study of 4,687 First-Year Medical Students From 49 LCME-Accredited U.S. Medical Schools

Measure (scale range)	Women (*n* = 2,344)				Men (*n* = 2,343)			
	Mean^*^ obese/overweight *n* = 378	Mean normal/under weight *n* = 1,966	b (SE)	p ^†^	Mean obese/overweight *n* = 768	Mean normal/under weight *n* = 1,575	b (SE)	p
Overall health (1–5)	3.35	3.74	−0.40 (0.04)	<0.001	3.60	3.89	−0.29 (0.03)	<0.001
Body esteem (1–5)	2.46	3.37	−0.91 (0.04)	<0.001	3.09	3.67	−0.57 (0.03)	<0.001
Social support (1–5)	4.11	4.23	−0.12 (0.03)	<0.001	4.01	3.99	0.02 (0.03)	0.49
Loneliness (1–5)	2.55	2.33	0.22 (0.04)	<0.001	2.33	2.37	−0.04 (0.03)	0.27
Mastery (1–7)	5.58	5.60	−0.02 (0.04)	0.67	5.76	5.68	0.08 (0.03)	0.02

* Means and parameter estimates adjusted for age, relationship status, non-white race, parents’ highest education, and social desirability

^†^ *P* values from Wald F tests

Higher scale scores represent more of the concept being measured

b beta coefficient, SE standard error

### Perceived Stigma and Self-Stigma

Overweight and obese students reported more perceived stigma during their lifetime than underweight/normal-weight students (scale mean difference = 0.12; *p* < 0.001). These associations did not vary significantly between men and women. A substantial proportion of overweight/obese students displayed some degree of explicit self-stigma. At least one item on each of the Anti-Fat Attitudes subscales was endorsed, including dislike of fat people (*n* = 268, 24 %), willpower (*n* = 758, 67 %), and "fear of fat" (*n* = 976, 87 %). Implicit self-stigma was common in the sample (*n* = 561) that completed the Fat-Thin IAT. Anti-fat implicit bias was demonstrated by 69 % (*n* = 389) of overweight/obese students, with 53 % (*n* = 298) exhibiting moderate or strong anti-fat bias.

### Perceived Stigma, Self-Stigma, and Stress Vulnerability Factors

In multivariate regression models including only students whose BMI was in the overweight/obese range, adjusted for age, gender, relationship status, race, parents’ education, and social desirability, perceived stigma was associated with all of the stress vulnerability factors (Table 4).

**Table 4:** Beta Coefficients (b) and Standard Errors (SE) for Linear Associations Between Discrimination/Self-Stigma and Stress Vulnerability Factors among 1,146 Obese/Overweight Students in the Medical Student CHANGES Study, a 2010 Web-Based Survey Study of First-Year Medical Students from 49 LCME-Accredited U.S. Medical Schools

Measure (scale range)	Perceived stigma (1–7)_ b (SE) *n* = 1,146	Explicit self-stigma b (SE) (1–7) *n* = 1,146			Implicit self-stigma b (SE) (−2 to 2) *n* = 561
		Dislike	Willpower	Fear	
Overall health (1–5)	−0.11 (0.03)‡§	0.01 (0.02)	0.04 (0.02)*^∥^	−0.11 (0.01) ‡	0.06 (0.06)
Anxiety (1–5)	0.18 (0.03) ‡	0.06 (0.01)†	−0.01 (0.01)	0.15 (0.02) ‡	−0.06 (0.06)
Depression (1–5)	0.20 (0.03) ‡	0.07 (0.01) ‡	−0.03 (0.02)	0.13 (0.01) ‡	−0.07 (0.07)
Fatigue (1–5)	0.13 (0.03) ‡	0.03 (0.02)	−0.01(0.01)	0.10 (0.01) ‡	−0.02 (0.07)
State self-esteem: confidence in cognitive abilities subscale (1–5)	−0.11 (0.03) ‡	−0.03 (0.02)	0.02 (0.01)	−0.10 (0.01) ‡	−0.04 (0.06)
State self-esteem: body esteem subscale (1–5)	−0.07 (0.03)*	0.01 (0.02)	0.01 (0.01)	−0.25 (0.01) ‡	−0.16 (0.06)*
State self-esteem: impression on others subscale (1–5)	−0.09 (0.04)*	−0.07 (0.02) ‡	0.02 (0.01)	−0.15 (0.02) ‡	−0.03 (0.07)
Mastery (1–7)	−0.25 (0.03) ‡	−0.10 (0.02) ‡	0.05 (0.02)†	−0.15 (0.01) ‡	0.08 (0.07)
Social Support (1–5)	−0.21 (0.03) ‡	−0.07 (0.02) ‡	0.04 (0.01)*	−0.06 (0.01) ‡	0.11 (0.06)
Loneliness (1–5)	0.26 (0.03) ‡	0.06 (0.02) ‡	0.01 (0.02)	0.15 (0.01) ‡	−0.11 (0.05)*
Use alcohol/drugs to cope (1–4)	0.10 (0.03)†	0.05 (0.01) ‡	0.01 (0.02)	0.05 (0.01) ‡	−0.04 (0.06)

*p < 0.05

^†^ *p* < 0.01, ‡ *p* < 0.001

§Adjusted for age, gender, relationship status, non-white race, parents’ highest education, and social desirability

^∥^ *P* values from Wald F tests

Higher scale scores represent more of the concept being measured

Among the explicit self-stigma measures, explicit dislike of fat people was associated with anxiety, depression, the importance of impression on others subscale of the self-esteem scale, sense of mastery, social support, loneliness, and use of alcohol or drugs to cope with stress. A stronger belief that willpower is a major determinant of obesity was associated with better overall health, a greater sense of mastery, and more social support. Fear of fat was strongly associated with all of the stress vulnerability factors. Implicit self-stigma was associated with lower body esteem, less loneliness, and less perceived stress.

## DISCUSSION

Overweight and obese medical students had significantly worse overall health and lower body esteem than normal-weight/underweight students, and were more likely to use drugs or alcohol to cope with stress, placing them at elevated risk for stress, which in turn could increase the risk of professional burnout,^48^ lower professionalism and empathy,^49^,^50^ lower academic performance,^49^,^51^–^53^ poor mental health,^49^,^54^ substance abuse,^55^ and suicidal ideation.^56^ Female students who were overweight/obese also reported less social support and more loneliness than their normal/underweight counterparts, suggesting that these students are more prone to the isolation and lack of support structure that impair health and predict burnout.^57^–^60^ Overweight/obese men (but not women) felt a greater sense of mastery, a psychological coping resource. One possible explanation for this finding is that men, but not women, who are overweight/obese must achieve a higher level of mastery than their peers in order to be competitive in medical school. More work is needed to understand these gender differences.

Students who were overweight/obese perceived more stigma, even after adjusting for race, gender, socioeconomic status, and age. Moreover, among overweight/obese students, perceived stigma was almost universally associated with conditions known to increase the risk of stress and reduce the ability to effectively cope with stress. This finding is noteworthy given the documented pressure of medical school and the link between stigma and risk of stress-related chronic diseases.^15^,^61^ In addition, evidence that physicians and trainees exhibit high levels of prejudice against individuals who are obese^27^,^62^–^64^ suggests that medical school may be especially threatening and stressful to overweight and obese students. However, first-year medical students who were overweight/obese had similar anxiety and depressive symptoms, self-esteem, and sense of mastery as their normal/underweight counterparts. This may be due to the universally heightened uncertainty that students experience during the first semester of medical school, which may have obscured group differences.

We found high levels of implicit and explicit self-stigma among overweight/obese students, among whom 70 % implicitly associated greater body size with negative words, suggesting internalization of these beliefs. Explicit self-stigma was associated with several stress vulnerability factors, although the strength of association varied among self-stigma components. Fear of gaining weight was strongly associated with each stress vulnerability measure. Dislike of fat people was also associated with greater depression and anxiety and lower self-esteem. The high prevalence of these attitudes suggests that the explicit in-group bias that can protect members of some minority groups may not apply for this group. Furthermore, as medical students and members of a generation exposed to public health messages touting the “war on obesity,” they may be especially likely to view obesity as a dangerous risk factor. This belief, though consistent with experience and social norms, is inconsistent with the need to maintain self-esteem—a conflict with negative implications for health and well-being.

Blaming people for being fat was not consistently associated with stress-related factors. The few associations with blame that reached significance were in a direction suggesting greater resiliency. This is surprising, given evidence that controllability beliefs are predictors of prejudice.^65^ It may be that the belief that weight is modifiable through willpower reinforces a sense of control over one’s own weight and protects against a feeling of helplessness. Another possibility, consistent with the system justification theory^66^, is that assimilation to the majority view of the causes of obesity increases one’s sense of belonging in medical school and common group identity with other students. This may also explain why implicit self-stigma was associated with less loneliness and perceived stress. Further study is warranted to examine whether perceived controllability of weight and implicit self-stigma are protective in some cases.

Implicit self-stigma was highly prevalent among overweight/obese students, suggesting that there is little automatic in-group bias in this population. Given the fact that in-group bias may protect self-esteem against the effects of stigma, the strong pro-thin implicit preferences found here are striking. Surprisingly, implicit self-stigma was not associated with worse well-being.

The primary limitation of this research is the cross-sectional nature of these data, which preclude inferences of causality. The focus of the survey on first-year students limits the generalizability of the findings to advanced trainees. However, first-year students represent a critical and vulnerable point^3^ in the progression toward a medical career, and thus are important to study in their own right. The current findings provide evidence that this population may require supportive services to prevent further degradation of well-being. Longitudinal study is needed to assess whether differences persist throughout medical school, and whether the trajectory of change for other factors differs according to BMI. Although we selected items from two scales that were representative of the concepts measured, we note that using selected items may affect measure validity. Also, many statistically significant associations have small effect sizes, and the clinical significance of these effects is difficult to assess. However, small differences that occur systematically may be meaningful, especially if those differences grow over time. Other limitations include the lack of direct measurement of student health or height/weight. However, the data were collected in a large national sample of medical students using validated measures, and provide novel insights regarding self-stigma and well-being among obese and overweight medical students.

We hypothesized that medical students who were overweight or obese—and female students in particular—have worse physical/mental health, fatigue, self-esteem, social support, and sense of mastery than non-overweight students. We found evidence to support this hypothesis for some but not all factors. We further hypothesized that among these students, those who have experienced more stigma or who are self-stigmatized will have worse outcomes. This hypothesis was supported by the data, with variation in experiences and attitudes associated with each factor. Our findings suggest that stigmatizing experiences may erode these students’ ability to cope with stress.

Medical schools should consider ways to support this group of students. Strategies that foster a non-threatening environment include enforcing zero tolerance of derogatory comments about overweight/obese patients, ensuring that curricula emphasize complex multifactorial causes of obesity, and including obesity and other stigmatized conditions in discussions about bias and disparities in care. To support students, schools could provide counselors trained to identify the effects of stigma on students’ ability to cope with stress, promote student awareness of the effects of stress on physical and mental health, and help students learn adaptive ways to regulate emotions. Additional qualitative research or more systematic information about programs to address the stigma of being overweight would complement the survey results from the present study in a valuable way. Effective strategies and interventions will benefit stigmatized students as well as the overall student body.
